# Lifestyle factors and colorectal cancer prediction: A nomogram-based model

**DOI:** 10.1186/s12885-025-14674-z

**Published:** 2025-07-29

**Authors:** Wooin Seo, Se Young Jung, Yeonhoon Jang, Kiheon Lee

**Affiliations:** 1 Office of Hospital Information, Seoul National University Bundang Hospital, Seongnam, Republic of Korea; 2 Department of Family Medicine, Seoul National University Bundang Hospital, Seongnam, Republic of Korea; 3 Department of Family Medicine, College of Medicine, Seoul National University, Seoul, Republic of Korea; 4 Department of Family Medicine, Seoul National University Bundang Hospital, 172 Dolma-ro, Bundang-gu, Seongnam-si, 13620 Republic of Korea

**Keywords:** Colorectal cancer, Nomograms, Lifestyle, Risk factors

## Abstract

**Background:**

Lifestyle factors are important contributors to the risk of colorectal cancer (CRC). This study developed and validated an age-based CRC risk-prediction model incorporating lifestyle factors using the National Health Insurance Service (NHIS)-National Sample Cohort database.

**Methods:**

Individuals who underwent the National Health Examination between 2009 and 2012 were eligible as study participants. Among them, 119,700 (30.38%) were aged 20–39, 190,645 (48.39%) were aged 40–59, and 83,611 (21.22%) were aged ≥ 60. Using the LASSO regression algorithm, we selected risk factors and fitted a Cox proportional hazards model to predict the 10-year CRC incidence. Nomogram-based risk scores were calculated for each age group. Candidate risk factors included sex, age, abdominal obesity, BMI, smoking, alcohol consumption, physical activity, presence of abnormal liver function, hypertension, hypercholesterolemia, and type 2 diabetes mellitus.

**Results:**

In each age group, higher risk scores had a higher incidence probability of CRC. In the discriminatory analysis, the concordance indices of the three models ranged from 0.60 to 0.70, indicating moderate discrimination power. The calibration plot from 10-fold cross-validation showed that the observed proportions of events and predicted probabilities overlapped the entire range of probabilities fairly well. Kaplan-Meier plots demonstrated that individuals in the high-risk group were more likely to develop CRC within 10 years than those in the low-risk group.

****Conclusion**:**

Lifestyle factors were identified as significant predictors of CRC incidence, with slight differences across age groups. Nomogram-based risk scores could be used as a tailored intervention to motivate individuals to modify their lifestyles.

**Supplementary Information:**

The online version contains supplementary material available at 10.1186/s12885-025-14674-z.

## Introduction

Colorectal cancer (CRC) is a major global health concern, ranking as the third leading cause of cancer-related deaths worldwide, with approximately 1.85 million new cases and 850,000 deaths annually [1]. In South Korea, CRC is one of the most commonly diagnosed cancers, with mortality ranking third among men and second among women [2]. Numerous studies have sought to quantify CRC risk through various scoring and stratification methods (S1 Table). Among these, nomogram-based models are widely used due to their graphical interface and ability to provide individualized risk estimates, facilitating personalized clinical decision-making [3, 4].

Previous CRC nomogram studies have primarily focused on survival outcomes—such as recurrence-free or overall survival—among patients already diagnosed with CRC [4]. Of 28 reviewed studies, only three targeted CRC risk prediction, and these were limited by small hospital-based samples and a focus on clinicopathological or treatment-related factors. Lifestyle and preventable risk factors were insufficiently addressed, and only one study included both validation and calibration analyses, underscoring the need for better-validated models using large population data.

While several studies have developed lifestyle-based CRC risk scores, none utilized a nomogram approach. Most applied logistic regression in cross-sectional designs, limiting predictive validity. Typical factors included smoking, alcohol use, physical activity, and dietary patterns (S1 Table), but few addressed age-specific variations in risk profiles. This gap highlights the importance of developing models that reflect how combinations of lifestyle and clinical risk factors vary across age groups.

Primary prevention of CRC remains essential [5], especially given strong evidence linking modifiable factors such as smoking, alcohol use, inactivity, and obesity to increased CRC risk [6–8]. Conditions like type 2 diabetes [9], hypertension [10], and abdominal obesity with hypercholesterolemia [11] have also been identified as significant risk factors. Liver function markers such as ALT and AST are increasingly recognized as CRC-related biomarkers, especially in individuals with obesity or fatty liver [12, 13].

Given the distinct epidemiological patterns of early-onset (< 50 years) and late-onset CRC (≥ 50 years) [5, 14], and the rising incidence in younger adults [15], guidelines have shifted—such as the American Cancer Society’s recommendation to begin CRC screening at age 45 [16]. However, few models have examined age-specific CRC risks in younger adults [17, 18]. Therefore, this study aims to develop and validate a lifestyle-based nomogram model for CRC risk stratified by three age groups (20–39, 40–59, and ≥ 60), using large-scale, population-based cohort data from South Korea.

## Materials and methods

### Study population

This study utilized data from the National Health Insurance Service–National Sample Cohort (NHIS-NSC), which includes a 2.2% representative sample (approximately 1 million individuals) of the Korean population enrolled between 2002 and 2003. The NHIS database covers 97% of the population and provides comprehensive information, including insurance claims, eligibility, death records, and biennial health screenings [19].

**Fig. 1:**
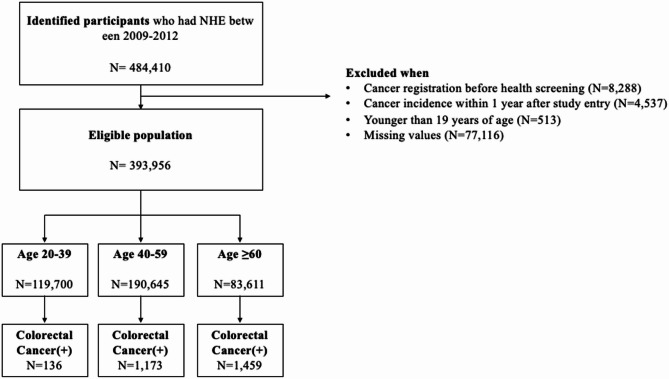
Flowchart of study population configuration

We included individuals who received at least one health screening between 2009 and 2012 (*n* = 484,410). Exclusion criteria were: age < 19 years (*n* = 513), prior cancer diagnosis before the health exam (*n* = 8,288), cancer diagnosis within one year of the exam (*n* = 4,537), and missing data in health screening variables (*n* = 77,116), leading to a final sample of 393,956 individuals (Fig. 1).

Diagnosis data were identified using ICD-10 codes from claims and reimbursement records. Lifestyle, anthropometric, and laboratory data were obtained from the national health screening program. To develop age-specific risk models, participants were categorized into three groups: 20–39 years (30.4%), 40–59 years (48.4%), and ≥ 60 years (21.2%).

### Variable definition

#### Risk factors

We used data from the Korean NHIS database, collecting risk factors at baseline and within one month of health screening (2009–2012). Based on prior evidence, we selected 11 CRC-related risk factors and stratified the study population into three age groups: 20–39, 40–59, and ≥ 60 years. In each group, age was dichotomized into younger (20s, 40s, 60s) and older (30s, 50s, ≥ 60) segments.

Obesity and overweight were defined using BMI thresholds of ≥ 25 kg/m² and ≥ 23 kg/m², respectively, per Korean guidelines [20]. Abdominal obesity was defined as waist circumference ≥ 90 cm in men and ≥ 85 cm in women [20].

Health behaviors were assessed via a standardized questionnaire before screening. Smoking was categorized by daily cigarette intake: light (< 10), moderate (10–19), and heavy (≥ 20) [21]. Alcohol intake was classified as none, normal, or heavy, with heavy drinking defined as > 2 times/week and > 7 drinks (men) or > 5 drinks (women) per session [22]. Physical activity levels were categorized into four groups using metabolic equivalent tasks (METs) with cutoffs at 500, 1000, and 1500 [23]. Although self-reported, these variables have been validated in prior NHIS-based studies.

Clinical conditions included type 2 diabetes, hypertension, abnormal liver function, and hypercholesterolemia. Diabetes was identified via fasting glucose ≥ 126 mg/dL or ICD-10 codes E11–E14 with medication records [24]. Hypertension was defined by systolic BP ≥ 140 mmHg, diastolic BP ≥ 90 mmHg, or relevant ICD-10 codes (I10–I13, I15) with prescriptions [24]. Abnormal liver function was indicated by serum glutamic oxaloacetic transaminase (SGOT) or serum glutamic pyruvate transaminase (SGPT) ≥ 40 IU/L [25]. Hypercholesterolemia was defined as total cholesterol ≥ 250 mg/dL or prescription of lipid-lowering medications [26].

#### Outcome

This study aimed to predict the 10-year incidence of CRC in patients who underwent health examinations between 2009 and 2012. The incidence of CRC was defined using the diagnostic codes for CRC (ICD-10 codes C18, C19, C20, and C21) from the claims database. The monitoring period for event occurrence was from baseline screening until December 31, 2019. In the main analysis, the outcome variable was the time between the date of the health examination and the date of the first CRC diagnosis or follow-up termination, whichever came first. Using individual information on the time of the CRC event, we calculated the 10-year incidence probability and CRC event-free survival rate to assess the validity of the nomogram-based scores.

### Statistical analysis

#### Descriptive analysis

We summarized demographic and clinical characteristics using mean ± SD for continuous variables and frequencies (%) for categorical variables. Student’s t-tests and Chi-squared tests were used to compare characteristics by CRC incidence, both in the total population and across age groups.

#### Feature selection and development of a risk model for CRC

Based on prior studies, 11 categorical variables were included as potential CRC risk factors: sex, age, abdominal obesity, BMI, smoking, alcohol consumption, physical activity, liver function, hypertension, hypercholesterolemia, and diabetes mellitus. To select features for each age group model, we applied LASSO-penalized Cox regression, which shrinks insignificant coefficients to zero [27]. Feature selection was performed using 10-fold cross-validation via the ‘cv.glmnet’ function in the R package ‘glmnet’. Hazard ratios (HR) and 95% confidence intervals (CI) were estimated using univariable and multivariable Cox models with the ‘survival’ package.

#### Development of nomogram-based risk scores for CRC prediction

Nomograms were developed using the coefficients from Cox regression. Each variable’s contribution was converted into a 0–100 point scale, with total scores representing the sum of individual points. Higher scores reflected increased CRC risk. Nomogram visualization was supported by the ‘rms’ package in R.

#### Validation of the nomogram-based risk prediction model

We assessed model performance using both discrimination and calibration. Discrimination, the model’s ability to distinguish between those with and without events, was measured by the concordance index (C-index). A C-index of 0.70–0.80 indicates adequate discrimination [28]. We reported both apparent and optimism-corrected C-index values based on 1000 bootstrap replications.

Calibration was evaluated by comparing predicted probabilities with observed CRC incidences using calibration plots. These plots, generated via 10-fold cross-validation using the ‘hdnom’ package, compared predicted 10-year risk to actual outcomes [4, 29].

We determined the optimal cut-off point for total risk scores using the ‘surv_cutpoint’ function in survminer [30]. This threshold maximized the separation between high- and low-risk groups based on 10-year CRC incidence. CRC-free survival rates between groups were compared using Kaplan-Meier curves. All statistical analyses were performed using R version 4.3.0, with significance set at *p* < 0.05.

## Results

### Demographic and clinical characteristics of the study population

Among the 393,956 eligible participants, 119,700 (30.38%) were aged 20 to 39, 190,645 (48.39%) were aged 40 to 59, and 83,611 (21.22%) were aged ≥ 60 (Table 1). Between January 1, 2009, and December 31, 2012, a total of 136 individuals aged 20–39 years, 1,173 individuals aged 40–59 years, and 1,459 individuals aged ≥ 60 years developed CRC.

**Table 1:** Demographic and clinical characteristics of the study population

	Study population (*N* = 393,956)		
	Colorectal cancer		p-value^a)^
	No	Yes	
N	391,188	2,768	
Age (years)			< 0.001
20–39	119,653 (30.6)	136 (4.9)	
40–59	189,472(48.4)	1173 (42.4)	
≥60	82,152 (21.0)	1459 (52.7)	
Sex (male)	170,899 (43.7)	1407 (50.8)	< 0.001
BMI			< 0.001
Normal and underweight	175,657 (44.9)	973 (35.2)	
Overweight	91,806 (23.5)	712 (25.7)	
Obesity	129,725 (31.6)	1083 (39.1)	
Smoking			0.002
None	280,490 (71.7)	1,961 (70.8)	
Light	13,247 (3.4)	88 (3.2)	
Moderate	47,019 (12.0)	300 (10.8)	
Heavy	50,432 (12.9)	419 (15.1)	
Alcohol drinking			< 0.001
None	219,813 (56.2)	1,665 (60.2)	
Normal	116,773 (29.9)	701 (25.3)	
Heavy	54,602 (14.0)	402 (14.5)	
Abdominal obesity (yes)	112,002 (28.6)	1,116 (40.3)	< 0.001
Physical activity			0.162
METs ≥ 1500	18,243 (4.7)	149 (5.4)	
METs < 1500	37,949 (9.7)	269 (9.7)	
METs < 1000	107,133 (27.4)	720 (26.0)	
METs < 500	227,863 (58.2)	1,630 (58.9)	
Hypertension (yes)	80,017 (20.5)	1,028 (37.1)	< 0.001
Hypercholesterolemia (yes)	45,306 (11.6)	468 (16.9)	< 0.001
Diabetes mellitus (yes)	27,874 (7.1)	394 (14.2)	< 0.001
Abnormal liver function (yes)	46,547 (11.9)	381 (13.8)	0.003
Age (years)	47.01 ± 14.39	59.55 ± 12.19	< 0.001
Waist circumference (cm)	79.38 ± 9.34	82.77 ± 8.67	< 0.001
Weight (kg)	62.66 ± 11.71	62.71 ± 10.68	0.825
BMI (kg/m2)	23.61 ± 6.02	24.22 ± 3.23	< 0.001
FPG (mg/dL)	97.04 ± 24.05	103.80 ± 30.48	< 0.001
Total cholesterol (mg/dL)	194.83 ± 41.17	200.31 ± 38.05	< 0.001
HDL-cholesterol (mg/dL)	56.71 ± 27.88	55.48 ± 28.27	0.021
LDL-cholesterol (mg/dL)	116.97 ± 141.18	117.33 ± 45.27	0.895
Systolic BP (mmHg)	121.73 ± 15.34	127.85 ± 16.22	< 0.001
Diastolic BP (mmHg)	75.75 ± 10.18	78.34 ± 9.98	< 0.001
SGOT (U/L)	24.93 ± 19.55	26.64 ± 15.77	< 0.001
SGPT (U/L)	24.31 ± 27.73	25.05 ± 16.20	0.164
GGT (U/L)	34.57 ± 48.90	43.31 ± 62.92	< 0.001
GFR (ml)	88.37 ± 30.92	77.63 ± 27.38	< 0.001

^a^P value are from Wilcoxon test for continuous variables and Pearson’s chi-squared test for categorical variables.

BMI, body mass index; METs, Metabolic Equivalents of Task; FPG, fasting plasma glucose; BP, blood pressure; SGOT, Serum Glutamic Oxaloacetic Transaminase; SGPT, Serum Glutamic Pyruvate Transaminase; GGT, Gamma-glutamyltransferase; GFR, Glomerular filtration rate.

Compared with individuals without CRC, those who developed cancer were older and had higher waist circumference (82.77 ± 8.67 vs. 79.38 ± 9.34), BMI (24.22 ± 3.23 vs. 23.61 ± 6.02), fasting plasma glucose (103.80 ± 30.48 vs. 97.04 ± 24.05), total cholesterol (200.31 ± 38.05 vs. 194.83 ± 41.17), and blood pressure (systolic blood pressure: 127.85 ± 16.22 vs. 121.73 ± 15.34; diastolic blood pressure: 78.54 ± 9.98 vs. 75.75 ± 10.18) at baseline. For categorical variables, individuals who developed cancer had higher proportions of obesity (39.1% vs. 31.6%), heavy smoking (15.1% vs. 12.9%), excessive alcohol consumption (14.5% vs. 14.0%), hypertension (37.1% vs. 20.5%), hypercholesterolemia (16.9% vs. 11.6%), type 2 diabetes mellitus (14.2% vs. 7.1%), and abnormal liver function (13.8% vs. 11.9%). The demographic and clinical characteristics of each age group are presented in S3 Table.

### Feature selection and development of a risk model for CRC

While the 11 candidate features were considered potential risk predictors, the LASSO regression algorithm determined which of the models under consideration was best. By computing the cross-validation error rate for each value of the tuning parameter (λ), we selected the λ with the minimum mean cross-validated error. After LASSO regression analysis, the age 20–39 cohort retained 7 variables with nonzero coefficients, removing BMI, abdominal obesity, alcohol drinking, and diabetes mellitus. For the age 40–59 cohort, 10 variables with nonzero coefficients were selected after excluding abnormal liver function. In the age ≥ 60 cohort, no variables were dropped from the model. The LASSO coefficient profiles for each model are presented in S1-3 Fig. We then fitted a Cox proportional hazards model with the selected risk factors to develop a nomogram for CRC prediction.

**Fig. 2:**
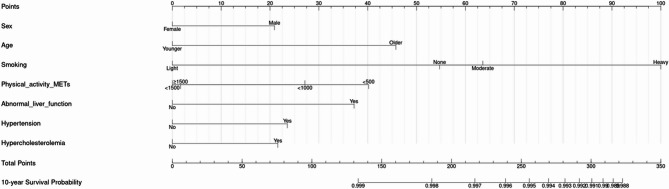
Nomogram for the prediction model of CRC incidence probability for age 20–39 group

**Fig. 3:**
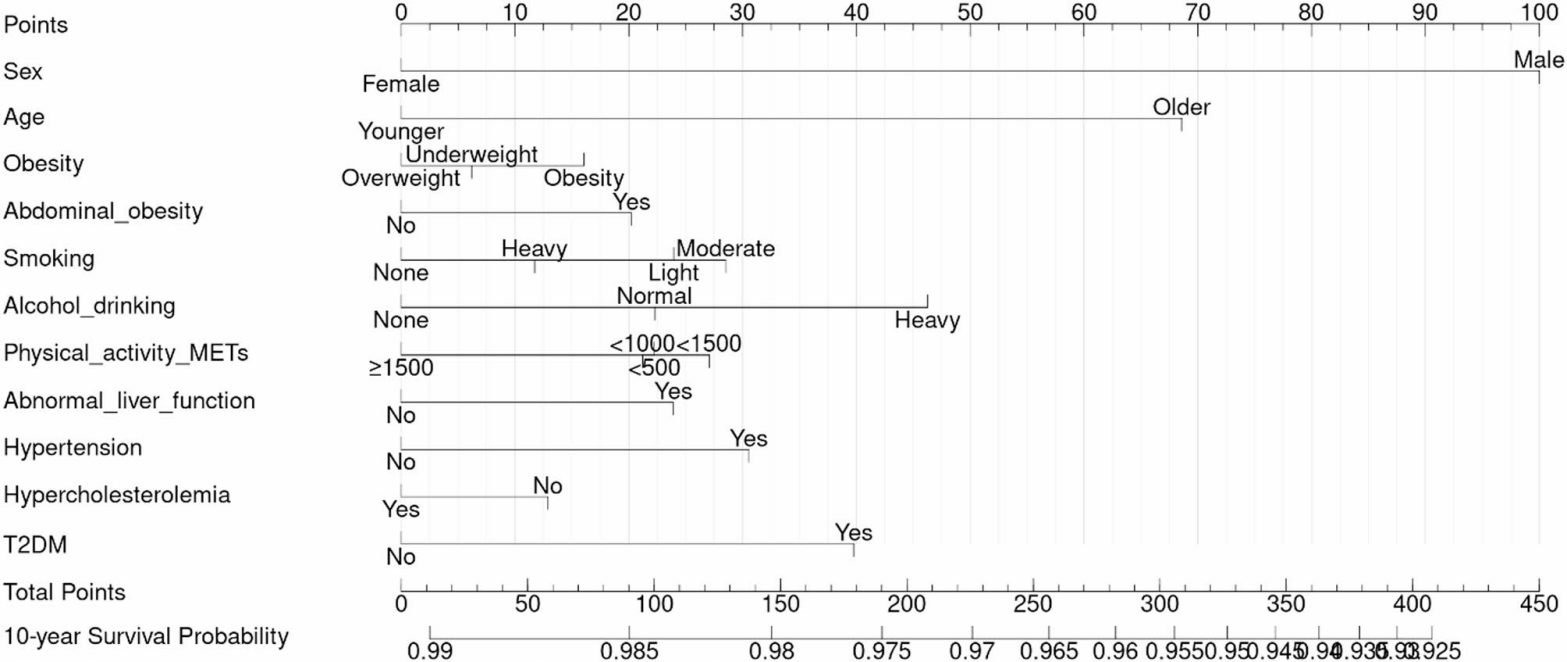
Nomogram for the prediction model of CRC incidence probability for age 40–59 group

**Fig. 4:**
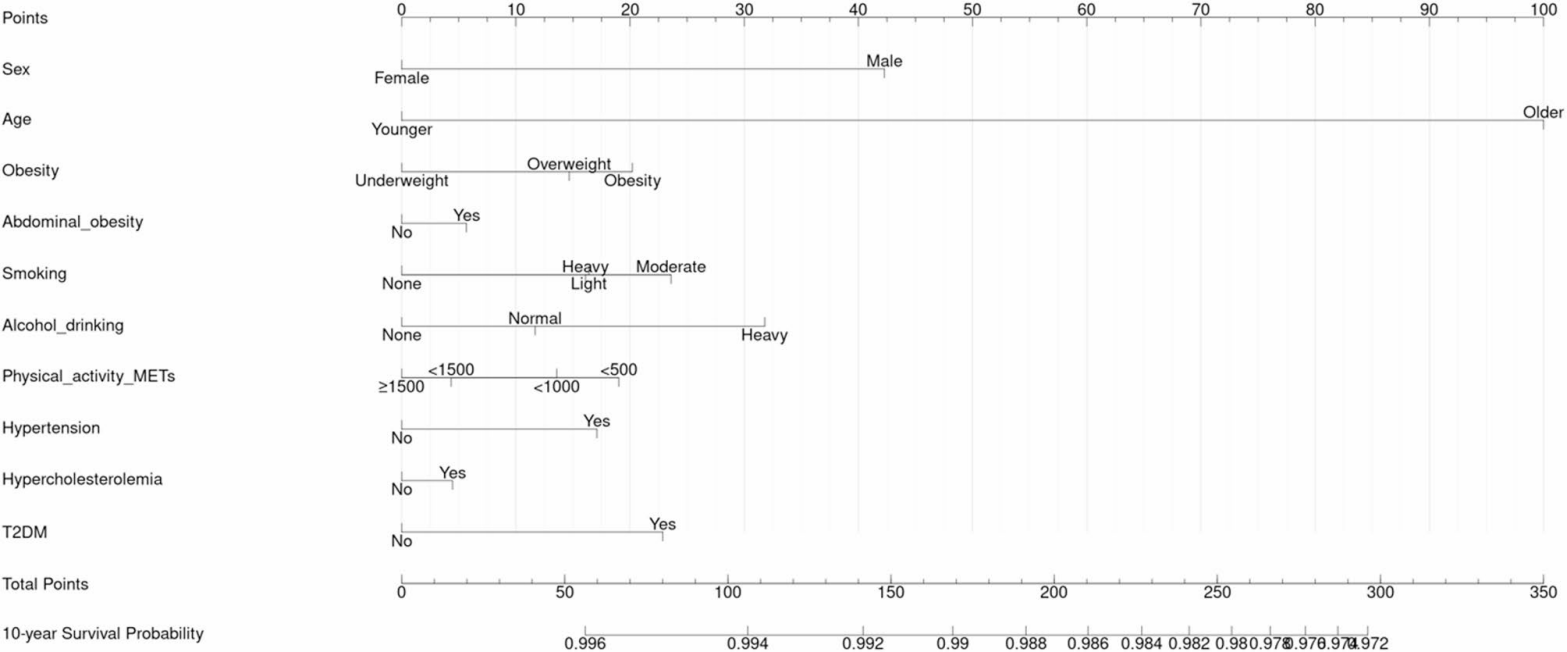
Nomogram for the prediction model of CRC incidence probability for age ≥ 60 group

The HRs of univariable and multivariable risk models are demonstrated in S4-6. Male sex and older age were significantly associated with increased CRC risk in every age group. In the 20–39 age group, heavy smoking and abnormal liver function were significantly associated with the risk of CRC incidence. For individuals in their 40 s and 50 s, alcohol drinking and hypertension had a significant association with CRC incidence. Although statistically less significant, obesity, moderate level of smoking, and the presence of diabetes mellitus were also identified as risk factors. In the model for age ≥ 60, heavy alcohol drinking, hypertension, and diabetes mellitus were associated with increased CRC risk. Using beta coefficients extracted from the Cox model, we constructed a risk score nomogram to estimate the 10-year risk of CRC incidence. The nomogram for the CRC prediction model is shown in Figs. 2, 3 and 4 and S7-S9 Table.

The C-index for CRC prediction was 0.684 in the age 20–39 cohort, 0.645 in the age 40–59 cohort, and 0.606 in the age ≥ 60 cohort. Across the 1000 bootstrap replications, the bootstrap optimism-corrected C-index for each age group was 0.657, 0.639, and 0.599, respectively.

### Development and validation of nomogram-based risk scores for CRC prediction

The total score was calculated as the sum of the scores for all input variables. The mean and standard deviation of the total score for each age cohort are reported in S10 Table. Since the variables included in the model and the scores for each risk factor category were constructed using a different logic by age group, total scores should be interpreted and compared within the same age group.

**Fig. 5:**
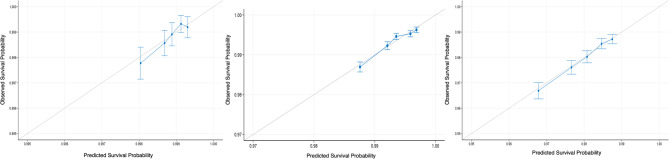
Calibration curve from 10-fold cross-validation depicting the correlation between the predicted and actual 10-year cancer event-free survival rates (Left: age 20–39; Middle: age 40–59; Right: age ≥ 60)

The calibration plot depicts the relationship between model-predicted and actual CRC development in the development and validation cohorts. A slope close to the 45° dashed line indicates that the predicted event-free survival rate corresponded well with the observed event-free survival rate. The calibration curve was produced by resampling internal data and conducting 10-fold cross-validation. In our three risk models, the observed proportions of events and predicted probabilities overlapped fairly well across the entire range of probabilities (Fig. 5).

**Fig. 6:**
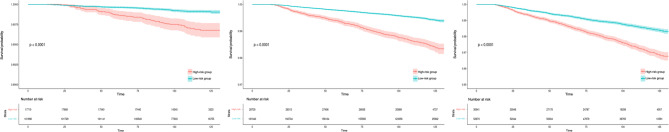
Kaplan-Meier curves of CRC for patients in high-risk and low-risk groups (Left: age 20–39; Middle: age 40–59; Right: age ≥ 60)

The optimal cut-off values of risk scores were 217, 177, and 162 for the age 20–39 group, the age 40–59 group, and the age ≥ 60 group respectively. Individuals were assigned to the low-risk or high-risk group based on the cut-off value for their respective age group. Among the age 20–39 group, 17,743 (14.82%) individuals were assigned to the high-risk group, and 101,957 (85.18%) individuals to the low-risk group. For the age 40–59 group, 28,705 (15.06%) individuals were identified as a high-risk group and 161,940 (84.94%) individuals as a low-risk group. In the age ≥ 60 group, there were 30,641 (36.65%) individuals at high risk, and 52,970 (63.35%) individuals at low risk. The Kaplan-Meier survival curve showed that the overall survival rate of the high-risk group was lower than that of the low-risk group, and the difference between them was statistically significant (*p* < 0.001) (Fig. 6).

## Discussion

In this study, we developed a nomogram-based CRC risk prediction model based on three age groups using the NHIS-NSC database. While previous studies have already discussed that risk factors for CRC varied by age group, few studies conducted variable selections and integrating them into risk scores across various age groups. Using the LASSO regression algorithm, we identified different sets of risk factors and constructed nomogram-based risk scores by age group. The risk scores not only quantified the relative importance of risk factors and their association with CRC incidence probability, but also had cut-off scores that effectively discriminated between high-risk and low-risk individuals. Our research findings from a nationally representative data could contribute to providing targeted interventions for modifiable risk factors.

In the age 20–39 group, heavy smoking was a dominant risk factor for CRC (Fig. 2). Smoking contributes to CRC development through mechanisms such as chronic inflammation, oxidative stress, and DNA damage [31]. Previous studies have shown that earlier initiation of smoking is associated with a higher CRC risk [32], and long-term heavy smokers tend to be diagnosed with CRC at a younger age compared to lifelong non-smokers [33]. These findings suggest that early CRC screening should be considered for young adults who smoke, given their elevated risk profile. Hypercholesterolemia was also selected as a risk factor in age 20–39 group, yet it ranked as the least important factor in the 40–59 age group and showed an inverse association in individuals aged 60 and older. In fact, more than half of hypercholesterolemia patients in South Korea receive lipid-lowering medications, particularly statins, with the majority being older patients [34]. This suggests that the lipid metabolic and CRC risk interactions could be age-dependent, with potential confounding factors such as prolonged statin therapy [35, 36].

The LASSO regression analysis identified lifestyle factors as more significant predictors of colorectal cancer than clinical conditions. Obesity-related risk factors were commonly observed, particularly among middle-aged individuals (ages 40–59), who demonstrated higher risk scores associated with elevated BMI levels. Overweight and obesity are well-known risk factors for CRC. Obesity contributes to the development of CRC through multiple mechanisms, including insulin resistance, chronic inflammation, and alterations in gut microbiota [11]. Therefore, weight management and lifestyle modifications, including dietary changes and regular physical activity, should be recommended as preventive measures. In our nomogram model, underweight status had the lowest score among adults aged 40–59, but scored higher than overweight status in those aged 60 and older. Since both obesity and underweight status are established risk factors for colorectal cancer [37], our findings suggest that underweight status becomes a more relevant risk factor in older age. Lack of physical activity was associated with an increased risk of CRC in both the 20–39 and 40–59 age groups. This finding aligns with previous research, which observed decreases in physical activity across all age groups and both sexes among incident CRC [5]. High-risk alcohol consumption, a statistically significant risk factor in both the 40–59 and age ≥ 60 groups, should also be considered a priority for intervention in CRC prevention. This finding is consistent with previous research pointing out that higher alcohol intake is associated with a significantly increased risk of CRC [38]. One possible mechanism linking alcohol consumption to CRC is its impairment of folate metabolism, as low folate levels have been associated with a 2- to 5-fold increased risk of CRC [39]. Therefore, limiting alcohol consumption should be a priority for middle-aged and elderly individuals at higher risk of CRC. Regarding the importance of age, the screening guideline based on the age of 50 was supported as robust in this study as well, as individuals in their 50s showed a significantly greater increase in risk compared to those in their 40s, having the largest HR (2.26, 95% CI: 2.00-2.55) compared with other models.

Our analysis may provide practical guidance by identifying an optimal cut-off for risk scores, indicating when the risk score significantly increases CRC risk. This may offer individuals insights into which risk factors among their combination of risks should be prioritized for management. For example, if a man aged 45 with obesity, abdominal obesity, and other underlying chronic conditions has even one of the three lifestyle risk factors, he would exceed the cut-off score of 111 and be classified as a high-risk group. Such score-based notifications would be effective in delivering an intuitive message to individuals about the necessity to reduce their risk factors.

The potential limitations of this study include recall bias and underestimation of the lifestyle (smoking, alcohol consumption, and physical activity) questionnaires. Second, other predictors causally associated with an increased risk of CRC were not included in the model because of limited data accessibility. Colonoscopy screening, for example, reduces CRC risk and plays a particularly important role in mitigating the risk associated with a family history of colorectal cancer [4]; however, data for colonoscopy history was not collected and nearly one-third of our study population (*n* = 133,766) had missing data on the family history of cancer. Besides, the questionnaire did not explicitly assess the family history of cancer, instead combining it with other diseases into a single question. Potential unobserved associations, such as individuals who have not undergone colonoscopy screening being more likely to have behavioral risk factors, may lead to an overestimated association between lifestyle factors and incident CRC in our study [40]. One study found that when family history factors (including a family history of gastrointestinal malignancy, cancer, or polyps) were incorporated into the risk model, smoking and alcohol consumption were not significant risk factors for CRC in individuals aged 25–49 [41]. Another study also revealed that when dietary factors were considered, heavy consumption of deep-fried foods had a greater impact on CRC risk than smoking and alcohol consumption [42]. Future studies that address these unobserved variables would ensure more robust analyses of lifestyle factors.

Third, the moderate discriminative power indicated by the C-index likely reflects the study’s limitations, including unobserved confounding factors and time-dependent effects of independent variables [43]. Although CRC is the type of cancer for which is known about the genes affected by cancer-causing mutations, genomic information was not available for this study. Additionally, the study design could not establish whether behavioral changes, positive or negative, led to changes in outcomes. If risk rankings become inconsistent over time, the model’s ability to differentiate between high- and low-risk individuals could decrease, resulting in a lower C-index [44]. The C-index was particularly lower in the older age group, suggesting potential unobserved confounders that were more predictive in late-onset CRC incidence [45]. While the calibration plot demonstrated good alignment between observed CRC incidence and predicted probabilities across the entire range, future research should include external validation using different population cohorts. Last but not least, since the study participants were limited to the Korean population, the results cannot be generalized to ethnic population groups with different CRC prevalence and incidence rates. Nevertheless, our results from a large, nationally representative sample will contribute to a basic investigation for future studies predicting CRC risk in other populations of different ethnicities.

Despite these limitations, to our knowledge, this is the first comprehensive study to develop a nomogram-based CRC risk model that includes and emphasizes lifestyle factors. Using a nationally representative sample, we obtained sufficient data to develop the risk models stratified by age groups. We expect that this study will provide a basis for future studies aimed at developing tailored interventions for CRC. In addition, it provides insights into future nomogram development for diseases with a large social burden, where individual preventive behavior is essential. This study also poses further research questions. Since the risk models contained individuals’ information from one point in time when they underwent a health examination, it is necessary to construct a risk model with time-dependent variables using repeatedly measured questionnaires. Furthermore, CRC risk prediction models should be more inclusive as they contain predictors such as genetic factors, family history, or colonoscopy if data collection is possible.

## Conclusions

Using nationally representative data, we developed nomogram-based risk models for CRC incorporating lifestyle factors. The LASSO regression algorithm identified distinct risk factors across different age groups (20–39, 40–59, and ≥ 60 years): younger adults (20–39 years) were primarily affected by behavioral risk factors such as smoking and alcohol consumption; middle-aged adults (40–59 years) showed stronger associations with obesity-related risk factors; and older adults (≥ 60 years) were most impacted by chronic conditions, particularly diabetes and hypertension. The total risk scores and established cut-off values provide practical guidance for targeted lifestyle modifications in CRC prevention across age groups.

## Electronic supplementary material

Below is the link to the electronic supplementary material.

Supplementary Material 1

## Data Availability

The data that support the findings of this study will be made available upon request and approval of a proposal by Korean National Health Insurance Service(https://nhiss.nhis.or.kr/).

## Abbreviations

ALT: Alanine aminotransferase
AST: Aspartate aminotransferase
BMI: Body mass index
BP: Blood pressure
C-index: Concordance index
CI: Confidence interval
CRC: Colorectal cancer
FPG: Fasting plasma glucose
GFR: Glomerular filtration rate
GGT: Gamma-glutamyltransferase
HR: Hazard ratio
LASSO: Least absolute shrinkage and selection operator
METs: Metabolic Equivalents of Task
NHIS-NSC: National Health Insurance Service-National Sample Cohort
SGOT: Serum Glutamic Oxaloacetic Transaminase
SGPT: Serum Glutamic Pyruvate Transaminase
