# Supplementary material for: Lifestyle factors and colorectal cancer prediction: A nomogram-based model
Source: BMC Cancer. 2025 Jul 29;25:1240. doi: 10.1186/s12885-025-14674-z (PMC12308952; doi:10.1186/s12885-025-14674-z)
Supplement: Supplementary file 1 — Supplementary Material 1 [file 12885_2025_14674_MOESM1_ESM.docx]

**S1 Table**. Previous research on risk scoring for colorectal cancer

| Author | Year | Country | Study setting | Number of participants | Method | Input features | Outcome | risk scoring method |
| --- | --- | --- | --- | --- | --- | --- | --- | --- |
| Imperiale et al. [1] | 2003 | United States | A company-based program of screening colonoscopy for  colorectal cancer | 1,994 | logistic regression | Age, sex, most advanced distal findings | risk for advanced proximal neoplasia | logistic regression for risk prediction → simple risk index→ risk score→ categorizing risk groups  (low/intermediate/high) |
| Ma et al. [2] | 2010 | Japan | Japan Public Health Center- based (JPHC) Prospective Study | 28,115 men for model development  18,256 men for model validation | cox hazard regression | Age, BMI, physical activity, family history of CRC, diabetes, alcohol consumption, smoking | risk for colorectal cancer | Cox hazard regression model → multiplying coefficients by three and then rounding them → simple point score model |
| Freedman et al. [3] | 2009 | United States | case-control study | 2,263 cases and 2,833 controls | logistic regression | Age, sex, sigmoidoscopy, colonoscopy, current leisure time activity, aspirin and NSAIDs, cigarette smoking, vegetables, BMI, hormone replacement, FDR with CRC, number of FDR with CRC | SEER incidence rates for proximal, distal, and rectal cancer | logistic regression → individual risk factor profiles → 10-year / 20-year absolute risk |
| Driver et al. [4] | 2007 | United States | Physician’s Health Study, a prospective cohort | 21,581 US male physicians | logistic regression | Age, alcohol use, smoking status, and body mass index | Predicted OR,  20-year Cumulative Risk of CRC | logistic regression → sum of the ORs of the final predictors → dividing into three risk class model |
| Steffen et al. [5] | 2014 | Australia | 45 and Up Study,  Melbourne Collaborative Cohort Study (MCCS) | 197,874 individuals for model development  24,233 for validation | proportional hazards regression | age, BMI, sex, prevalent diabetes, previous colorectal cancer screening, first-degree relative with colorectal cancer, aspirin use, smoking status, alcohol intake, dietary fiber intake, intake of vegetables, fruits, red meat and processed meat, and vigorous physical activity | 5-year  colorectal cancer risk | proportional hazard regression → risk score computed as linear combination of the weighted predictors |
| Wu et al. [6] | 2022 | China | CRC screening program in Shanghai | 807,695 subjects | logistic regression (LR)  artificial neural Network (ANN) | age, sex, chronic diarrhea, mucus or bloody stool, diagnosis of any cancer, CRC in first degrees relatives, Qualitive FIT | CRC in screening | establishing predictive model using LR and ANN approaches → calculating LR-based risk score using age-standardized method → ANN-based risk score computed by the LR-based risk score by contributions of predictors on the outcome in the ANN model |
| Jeon et al. [7] | 2018 | United States | Genetics and  Epidemiology of Colorectal Cancer Consortium (GECCO), Colorectal  Transdisciplinary study (CORECT) | 9,748 CRC cases and 10,590 controls | multivariate logistic regression | 19 lifestyle and environmental factors, 63 single-nucleotide polymorphisms, family history | CRC detection in screening | multivariate logistic regressions each for E-score and G-score → summing all the risk factors weighted by their log-odds ratio estimates |
| Carr et al. [8] | 2020 | Germany | Darmkrebs: Chancen der Verhütung durch Screening [DACHS]  study | 4,220 patients with CRC and 3,338 individuals without CRC | Multiple logistic regression | healthy lifestyle score (smoking, alcohol consumption, diet, physical activity, and body fatness), a polygenic risk score (based on 90 single-nucleotide polymorphisms), and colonoscopy history | 30-year absolute risk estimates for development of CRC | A healthy lifestyle score and polygenic risk score were calculated based on questionnaire and blood test |

**S2 Table.** Health checkup questionnaire in general health examination

| Smoking-Related Questions4. Please read the questions below and fill in the details corresponding to your current situation. 4-1. **Have you ever smoked at least 5 packs (100 cigarettes) in your lifetime?**   1. No (Proceed to Question 5). 2. Yes, but I have quit (Proceed to Question 4-2). 3. Yes, and I am currently smoking (Proceed to Question 4-3).   **4-2. If you used to smoke but have quit:**   - For how many years did you smoke? Total: ___ years - Before quitting, what was your average daily cigarette consumption? ___ cigarettes/day   **4-3. If you are currently smoking:**   - For how many years have you been smoking? Total: ___ years - What is your average daily cigarette consumption? ___ cigarettes/day |
| --- |
| Alcohol-Related Questions5. Please read the questions below and fill in the details corresponding to your current situation. 5-1. On **average, how many days per week do you drink alcohol?** □ 0 □ 1 □ 2 □ 3 □ 4 □ 5 □ 6 □ 7  **5-2. On days you drink alcohol, how much do you typically consume?** ( ___ glasses) (Regardless of the type of alcohol, calculate in terms of standard glasses. 1 can of beer (355 ml) is equivalent to 1.6 standard glasses.) |
| Physical Activity (Exercise)-Related Questions6. Please read the questions below and fill in the details corresponding to your current activity level in the past 1 week. 6-1. In the past 1 week, how many days did you engage in vigorous physical activities (e.g., running, aerobics, fast cycling, hiking, etc.) that made you breathe much harder than usual for at least 20 minutes per day? □ 0 □ 1 □ 2 □ 3 □ 4 □ 5 □ 6 □ 7  6-2. In the past 1 week, how many days did you engage in moderate physical activities (e.g., brisk walking, tennis, cycling at a regular speed, gardening, etc.) that made you breathe somewhat harder than usual for at least 30 minutes per day? □ 0 □ 1 □ 2 □ 3 □ 4 □ 5 □ 6 □ 7  (Note: Exclude any physical activities already reported in 6-1.)  6-3. In the past 1 week, how many days did you walk for at least 10 minutes at a time, totaling 30 minutes or more per day? (Include light exercises, leisure walking, or commuting walking.) □ 0 □ 1 □ 2 □ 3 □ 4 □ 5 □ 6 □ 7 |

**S3 Table.** Demographic and clinical characteristics of each age group

|  | **Age 20–39 (N=119,700**) | | | |  | **Age 40–59 (N=190,645)** | | | | | | |  |  | **Age ≥60 (N=83,611)** | | | | | | |
| --- | --- | --- | --- | --- | --- | --- | --- | --- | --- | --- | --- | --- | --- | --- | --- | --- | --- | --- | --- | --- | --- |
|  | Colorectal cancer | | | p-value^b)^ | | | Colorectal cancer | | | | p-value^b^ | | | | |  | Colorectal cancer | | | p-value^b^ | |
|  | No | Yes | |  |  |  | No | | Yes | |  |  |  |  |  |  | No | Yes | |  |  |
| N | 119,564 | | 136 |  | | |  | 189,472 | | 1,173 |  | | | | |  | 82,152 | | 1,459 |  | |
| Sex (male) | 66,797(55.9) | | 98(72.1) | <0.001 | | | 75,302(39.7) | | 621(52.9) | | | <0.001 | |  | | 28,800(35.1) | | | 688(47.2) | | <0.001 |
| BMI |  | |  | 0.433 | | |  | |  | | | <0.001 | |  | |  | | |  | | 0.104 |
| Normal and Underweight | 66,184(55.4) | | 68(50.0) |  | | | 79,941(42.2) | | 403(34.4) | | |  | |  | | 29,532(35.9) | | | 502(34.4) | |  |
| Overweight | 22,135(18.5) | | 27(19.9) |  | | | 47,981(25.3) | | 317(27.0) | | |  | |  | | 21,690(26.4) | | | 368(25.2) | |  |
| Obesity | 31,245(26.1) | | 41(30.1) |  | | | 61,550(32.5) | | 453(38.6) | | |  | |  | | 30,930(37.6) | | | 589(40.4) | |  |
| Smoking |  | |  | <0.001 | | |  | |  | | | <0.001 | |  | |  | | |  | | 0.104 |
| None | 73,215(61.2) | | 63(46.3) |  | | | 138,168(72.9) | | 747(63.7) | | |  | |  | |  | | |  | | <0.001 |
| Light | 6,105(5.1) | | 3(2.2) |  | | | 4,995(2.6) | | 35(3.0) | | |  | |  | | 69,107(84.1) | | | 1151(78.9) | |  |
| Moderate | 24,011(20.1) | | 31(22.8) |  | | | 18,236(9.6) | | 151(12.9) | | |  | |  | | 2,147(2.6) | | | 50(3.4) | |  |
| Heavy | 16,233(13.6) | | 39(28.7) |  | | | 28,073(14.8) | | 240(20.5) | | |  | |  | | 4,772(5.8) | | | 118(8.1) | |  |
| Alcohol drinking |  | |  | 0.101 | | |  | |  | | | <0.001 | |  | |  | | |  | | <0.001 |
| None | 45,964(38.4) | | 41(30.1) |  | | | 109,639(57.9) | | 593(50.6) | | |  | |  | | 64,210(78.2) | | | 1031(70.7) | |  |
| Normal | 50,659(42.4) | | 62(45.6) |  | | | 52,994(28.0) | | 344(29.3) | | |  | |  | | 13,120(16.0) | | | 295(20.2) | |  |
| Heavy | 22,941(19.2) | | 33(24.3) |  | | | 26,839(14.2) | | 236(20.1) | | |  | |  | | 4,822(5.9) | | | 133(9.1) | |  |
| Abdominal obesity (yes) | 18,659(15.6) | | 29(21.3) | 0.086 | | | 53,625(28.3) | | 379(32.3) | | | 0.003 | |  | | 39,718(48.3) | | | 708(48.5) | | 0.913 |
| Physical activity |  | |  | 0.361 | | |  | |  | | | 0.893 | |  | |  | | |  | | 0.864 |
| METs≥1500 | 3,901(3.3) | | 3(2.2) |  | | | 9,035(4.8) | | 54(4.6) | | |  | |  | | 5,307(6.5) | | | 92(6.3) | |  |
| METs<1500 | 10,743(9.0) | | 8(5.9) |  | | | 19,365(10.2) | | 113(9.6) | | |  | |  | | 7,841(9.5) | | | 148(10.1) | |  |
| METs<1000 | 36,295(30.4) | | 38(27.9) |  | | | 49,977(26.4) | | 308(26.3) | | |  | |  | | 20,861(25.4) | | | 374(25.6) | |  |
| METs<500 | 68,625(57.4) | | 87(64.0) |  | | | 111,095(58.6) | | 698(59.5) | | |  | |  | | 48,143(58.6) | | | 845(57.9) | |  |
| Hypertension (yes) | 7,801(6.5) | | 17(12.5) | 0.008 | | | 36,513(19.3) | | 307(26.2) | | | <0.001 | |  | | 35,703(43.5) | | | 704(48.3) | | <0.001 |
| Hypercholesterolemia (yes) | 4,823(4.0) | | 11(8.1) | 0.029 | | | 23,406(12.4) | | 177(15.1) | | | 0.005 | |  | | 17,077(20.8) | | | 280(19.2) | | 0.145 |
| Diabetes mellitus (yes) | 2,175(1.8) | | 3(2.2) | 0.987 | | | 13,576(7.2) | | 128(10.9) | | | <0.001 | |  | | 12,123(14.8) | | | 263(18.0) | | 0.001 |
| Abnormal liver function (yes) | 14,839(12.4) | | 34(25.0) | <0.001 | | | 23,465(12.4) | | 171(14.6) | | | 0.026 | |  | | 8,243(10.0) | | | 176(12.1) | | 0.012 |
|  |  | |  |  | | |  | |  | | |  | |  | |  | | |  | |  |
| Age (years) | 30.58±5.09 | | 32.90±4.57 | <0.001 | | | 48.29±5.68 | | 50.94±5.42 | | | <0.001 | |  | 67.96±6.23 | | | | 68.96±6.14 | | <0.001 |
| Waist circumference (cm) | 77.13±10.07 | | 80.34±10.09 | <0.001 | | | 79.40±8.82 | | 81.53±8.75 | | | <0.001 | |  | 82.63±8.40 | | | | 83.99±8.28 | | <0.001 |
| Weight (kg) | 65.17±13.67 | | 68.51±14.06 | 0.004 | | | 62.57±10.73 | | 64.62±10.62 | | | <0.001 | |  | 59.23±9.73 | | | | 60.64±9.86 | | <0.001 |
| BMI (kg/m2) | 22.94±3.65 | | 23.64±3.88 | 0.025 | | | 23.79±3.13 | | 24.22±3.19 | | | <0.001 | |  | 24.16±11.38 | | | | 24.26±3.18 | | 0.73 |
| FPG (mg/dL) | 90.77±17.60 | | 91.93±14.28 | 0.443 | | | 98.24±24.96 | | 102.16±30.46 | | | <0.001 | |  | 103.41±27.66 | | | | 106.22±31.25 | | <0.001 |
| Total cholesterol (mg/dL) | 183.88±35.55 | | 193.88±34.85 | 0.001 | | | 199.26±40.63 | | | 202.81±37.20 | | 0.003 | |  | 200.55±46.59 | | | | 198.89±38.89 | | 0.176 |
| HDL-cholesterol (mg/dL) | 58.14±26.43 | | 54.75±13.45 | 0.136 | | | 56.74±26.99 | | 56.09±29.21 | | | 0.41 | |  | 54.55±31.61 | | | | 55.05 ±28.51 | | 0.543 |
| LDL-cholesterol (mg/dL) | 112.30±29.84 | | 108.22±30.19 | 0.838 | | | 118.60±80.03 | | | 117.76±35.71 | | 0.72 | |  | 120.02±56.32 | | | | 117.82±52.60 | | 0.139 |
| Systolic BP (mmHg) | 117.30±13.17 | | 121.62±13.09 | <0.001 | | | 121.29±15.00 | | 123.92±15.21 | | | <0.001 | |  | 129.20±16.23 | | | | 131.59±16.35 | | <0.001 |
| Diastolic BP (mmHg) | 73.52±9.46 | | 76.32±8.93 | 0.001 | | | 76.12±10.37 | | 78.12±10.23 | | | <0.001 | |  | 78.16±10.12 | | | | 79.08±9.82 | | <0.001 |
| SGOT (U/L) | 23.05±18.97 | | 25.61±13.01 | 0.116 | | | 25.40±20.75 | | 26.37±18.31 | | | 0.113 | |  | 26.57±17.14 | | | | 26.96±13.65 | | 0.376 |
| SGPT (U/L) | 23.94±27.84 | | 28.95±22.87 | 0.036 | | | 24.94±26.07 | | 26.08±17.36 | | | 0.136 | |  | 23.40±31.04 | | | | 23.85±14.27 | | 0.58 |
| GGT (U/L) | 30.63±34.86 | | 46.18±50.61 | <0.001 | | | 37.43±55.12 | | 47.50±72.13 | | | <0.001 | |  | 33.69±50.66 | | | | 39.68±55.38 | | <0.001 |
| GFR (ml) | 102.75±31.89 | | 100.40±22.94 | 0.497 | | | 87.09±27.61 | | 86.31±21.87 | | | 0.457 | |  | 66.99±22.60 | | | | 67.36±28.10 | | 0.653 |
| ^a)^The first health examination record from 2009 set as the baseline. ^b)^P value are from Wilcoxon test for continuous variables and Pearson's chi-squared test for categorical variables. BMI, body mass index; METs, Metabolic Equivalents of Task; FPG, fasting plasma glucose; BP, blood pressure; SGOT, Serum Glutamic Oxaloacetic Transaminase; SGPT, Serum Glutamic Pyruvate Transaminase; GGT, Gamma-glutamyl transferase; GFR, Glomerular filtration rate. | | | | | | | | | | | | | | | | | | | | | |

**S4 Table.** Cox Regression Model for 10-year Incidence of CRC for age 20–39 group

|  |  | **Univariable model** | | |  | **Multivariable model** | | | | | | | |
| --- | --- | --- | --- | --- | --- | --- | --- | --- | --- | --- | --- | --- | --- |
|  |  | **HR** | **95% CI** | **p-value** | **C-index (se)** | **HR** | **95% CI** | | **p-value** | | | | |
| **Sex** | | 1.98 | 1.36-2.89 | <0.001 | 0.578 (0.02) | 1.31 | 0.80-2.14 | | 0.271 | | | | |
| **Age** | | 2.24 | 1.52-3.3 | <0.001 | 0.594 (0.01) | 1.82 | 1.23-2.70 | | 0.002 | | | | |
| **Smoking** | |  |  |  | 0.61 (0.02) |  | |  | |  |  |  |  |
|  | None | 1 |  |  |  | 1 |  | |  | | | | |
|  | Light | 0.57 | 0.18-1.82 | 0.346 |  | 0.48 | 0.15-1.57 | | 0.229 | | | | |
|  | Moderate | 1.47 | 0.96-2.27 | 0.075 |  | 1.12 | 0.67-1.85 | | 0.649 | | | | |
|  | Heavy | 2.72 | 1.83-4.06 | <0.001 |  | 1.81 | 1.11-2.94 | | 0.016 | | | | |
| **Physical activity (METs)** | |  |  |  |  |  | |  | |  |  |  |  |
|  | ≥1500 | 1 |  |  | 0.524(0.02) | 1 |  | |  | | | | |
|  | <1500 | 0.96 | 0.25-3.64 | 0.962 |  | 0.97 | 0.25-3.68 | | 0.973 | | | | |
|  | <1000 | 1.36 | 0.42-4.42 | 0.604 |  | 1.39 | 0.43-4.53 | | 0.577 | | | | |
|  | <500 | 1.64 | 0.52-5.21 | 0.394 |  | 1.65 | 0.52-5.25 | | 0.389 | | | | |
| **Abnormal liver function** | | 2.32 | 1.57-3.42 | <0.001 | 0.561 (0.01) | 1.63 | 1.07-2.47 | | | | 0.021 | | |
| **Hypertension** | | 2.03 | 1.22-3.38 | 0.012 | 0.526 (0.01) | 1.63 | 0.80-2.30 | | | | 0.249 | | |
| **Hypercholesterolemia** | | 2.07 | 1.11-3.83 | 0.020 | 0.522 (0.01) | 1.32 | 0.70-2.50 | | | | 0.383 | | |
|  |  |  |  |  | **C-index (se)** 0.684 | | | | | | |  |  |
|  |  |  |  |  | **Optimism-corrected c-index** 0.657 | | | | | | |  |  |

**S5 Table.** Cox Regression Model for 10-year Incidence of CRC for age 40–59 group

|  | |  | **Univariable model** | | | |  | | | **Multivariable model** | | |
| --- | --- | --- | --- | --- | --- | --- | --- | --- | --- | --- | --- | --- |
|  | |  | **HR** | **95% CI** | **p-value** | | **C-index (se)** | | **HR** | | **95% CI** | **p-value** |
| **Sex** | |  | 1.68 | 1.50-1.88 | | <0.001 | 0.565 (0.007) | | 1.41 | | 1.20-1.65 | <0.001 |
| **Age** | |  | 2.25 | 2.00-2.54 | | <0.001 | 0.602 (0.007) | | 2.26 | | 2.00-2.55 | <0.001 |
| **BMI** | |  |  |  | |  | 0.543 (0.008) | |  | |  |  |
|  | <23 kg/m2 | | 1 |  | |  |  | | 1 | |  |  |
|  | <25 kg/m2 | | 1.29 | 1.11-1.50 | | <0.001 |  | | 1.12 | | 0.96-1.31 | 0.119 |
|  | ≥25 kg/m2 | | 1.45 | 1.27-1.66 | | <0.001 |  | | 1.17 | | 0.99-1.39 | 0.051 |
| **Abnormal obesity** | |  | 1.21 | 1.07-1.37 | | 0.001 | 0.519 (0.007) | | 1.04 | | 0.89-1.22 | 0.554 |
| **Smoking** | | | 1 |  | |  | 0.546 (0.007) | | 1 | |  |  |
|  | None | |  |  | |  |  | |  | |  |  |
|  | Light | | 1.32 | 0.94-1.86 | | 0.102 |  | | 1.14 | | 0.80-1.62 | 0.450 |
|  | Moderate | | 1.53 | 1.29-1.83 | | <0.001 |  | | 1.21 | | 0.99-1.48 | 0.061 |
|  | Heavy | | 1.59 | 1.38-1.84 | | <0.001 |  | | 1.14 | | 0.94-1.37 | 0.161 |
| **Alcohol drinking** | | | |  | |  | 0.544 (0.008) | |  | |  |  |
|  | None | | 1 |  | |  |  | | 1 | |  |  |
|  | Normal | | 1.19 | 1.04-1.36 | | 0.007 |  | | 1.10 | | 0.95-1.27 | 0.193 |
|  | Heavy | | 1.63 | 1.40-1.89 | | <0.001 |  | | 1.29 | | 1.08-1.54 | 0.004 |
| **Physical activity (METs)** | | | |  | |  | 0.505 (0.008) | |  | |  |  |
|  | ≥1500 | | 1 |  | |  |  | | 1 | |  |  |
|  | <1500 | | 0.97 | 0.70-1.35 | | 0.895 |  | | 1.03 | | 0.74-1.43 | 0.830 |
|  | <1000 | | 1.03 | 0.77-1.38 | | 0.801 |  | | 1.11 | | 0.83-1.49 | 0.452 |
|  | <500 | | 1.06 | 0.80-1.39 | | 0.675 |  | | 1.16 | | 0.88-1.54 | 0.272 |
| **Hypertension** | | | 1.49 | 1.31-1.70 | | <0.001 | 0.535 (0.007) | | 1.14 | | 1.00-1.31 | 0.045 |
| **Hypercholesterolemia** | | | 1.27 | 1.08-1.49 | | 0.004 | 0.516 (0.005) | | 1.03 | | 0.88-1.22 | 0.663 |
| **Diabetes Mellitus** | | | 1.62 | 1.34-1.94 | | <0.001 | 0.519 (0.005) | | 1.20 | | 0.99-1.45 | 0.057 |
|  | |  |  |  | |  |  | | **C-index** 0.645 | | | |
|  | |  |  |  | |  |  | **Optimism-corrected c-index** 0.639 | | | | |

**S6 Table.** Cox Regression Model for 10-year Incidence of CRC for age ≥60 group

|  | |  | **Univariable model** | | | |  | | | **Multivariable model** | | |
| --- | --- | --- | --- | --- | --- | --- | --- | --- | --- | --- | --- | --- |
|  | |  | **HR** | **95% CI** | **p-value** | | **C-index (se)** | | **HR** | | **95% CI** | **p-value** |
| **Sex** | |  | 1.76 | 1.59-1.96 | | <0.001 | 0.567 (0.007) | | 1.67 | | 1.46-1.91 | <0.001 |
| **Age** | |  | 1.37 | 1.23-1.52 | | <0.001 | 0.538 (0.007) | | 1.42 | | 1.28-1.58 | <0.001 |
| **BMI** | |  |  |  | |  | 0.512 (0.007) | |  | |  |  |
|  | <23 kg/m2 | | 1 |  | |  |  | | 1 | |  |  |
|  | <25 kg/m2 | | 0.96 | 0.84-1.10 | | 0.565 |  | | 0.96 | | 0.84-1.11 | 0.656 |
|  | ≥25 kg/m2 | | 1.06 | 0.94-1.20 | | 0.283 |  | | 1.05 | | 0.90-1.22 | 0.507 |
| **Abnormal obesity** | | | 0.97 | 0.88-1.08 | | 0.672 | 0.5 (0.007) | | 1.11 | | 0.96-1.27 | 0.135 |
| **Smoking** | | | 1 |  | |  | 0.529(0.005) | |  | |  |  |
|  | None | | 1 |  | |  |  | | 1 | |  |  |
|  | Light | | 1.53 | 1.15-2.03 | | 0.003 |  | | 1.13 | | 0.84-1.51 | 0.408 |
|  | Moderate | | 1.59 | 1.32-1.93 | | <0.001 |  | | 1.15 | | 0.94-1.41 | 0.160 |
|  | Heavy | | 1.47 | 1.23-1.75 | | <0.001 |  | | 1.06 | | 0.87-1.29 | 0.541 |
| **Alcohol drinking** | | | |  | |  | 0.54 (0.006) | |  | |  |  |
|  | None | | 1 |  | |  |  | | 1 | |  |  |
|  | Normal | | 1.41 | 1.24-1.61 | | <0.001 |  | | 1.12 | | 0.97-1.29 | 0.115 |
|  | Heavy | | 1.76 | 1.47-2.11 | | <0.001 |  | | 1.26 | | 1.03-1.54 | 0.022 |
| **Physical activity (METs)** | | | |  | |  | 0.504 (0.007) | |  | |  |  |
|  | ≥1500 | | 1 |  | |  |  | | 1 | |  |  |
|  | <1500 | | 1.08 | 0.83-1.40 | | 0.54 |  | | 1.15 | | 0.88-1.49 | 0.292 |
|  | <1000 | | 1.04 | 0.83-1.31 | | 0.699 |  | | 1.11 | | 0.88-1.40 | 0.347 |
|  | <500 | | 1.03 | 0.83-1.28 | | 0.751 |  | | 1.12 | | 0.90-1.39 | 0.298 |
| **Abnormal liver function** | | | 1.27 | 1.08-1.48 | | 0.002 | 0.511 (0.004) | | 1.13 | | 0.96-1.32 | 0.132 |
| **Hypertension** | | | 1.23 | 1.11-1.36 | | <0.001 | 0.527 (0.007) | | 1.17 | | 1.05-1.30 | 0.003 |
| **Hypercholesterolemia** | | | 0.89 | 0.78-1.02 | | 0.096 | 0.508 (0.005) | | 0.93 | | 0.81-1.07 | 0.329 |
| **Diabetes Mellitus** | | | 1.32 | 1.16-1.51 | | <0.001 | 0.519 (0.005) | | 1.22 | | 1.07-1.40 | 0.003 |
|  | |  |  |  | |  |  | | **C-index** 0. 606 | | | |
|  | |  |  |  | |  |  | **Optimism-corrected c-index** 0.599 | | | | |

**S7 Table.** Scores for each risk factor category for age 20–39 group

| **Variable** | | **Categories and Scores** | | | | | | | | | | | | | | | | | | |  |  |  |
| --- | --- | --- | --- | --- | --- | --- | --- | --- | --- | --- | --- | --- | --- | --- | --- | --- | --- | --- | --- | --- | --- | --- | --- |
| 1. Sex | | **Female** | | | | | | **Male** | | | | |  |  | | | |  | | |  |  |  |
|  |  | 0 | | | | | | 21 | | | | |  |  | | | |  | | |  |  |  |
| 2. Age | | **20s** | | | | | **30s** | | | | |  | | |  | | |  | | |  |  |  |
|  |  | 0 | | | | | 46 | | | | |  | | |  | | |  | | |  |  |  |
| 3. Smoking | | **None** | | | | **Light** | | | | | | | **Moderate** | | | | **Heavy** | | | | |  |  |
|  |  | 55 | | 0 | | | | | | | 64 | | | | | | 100 | | | | |  |  |
| 4. Physical activity (METs) | | **≥1500** | | | | | | | **<1500** | | | | | **<1000** | | | | **<500** | |  | | | |
|  |  | 2 | | | | | | | 0 | | | | | 27 | | | | 40 |  | | | |  |
| 5. Abnormal liver function | | **No** | | | **Yes** | | | | | |  | | | | |  | |  | | |  |  |  |
|  |  | 0 | 37 | | | | | | |  | | | | | |  | |  | | |  |  |  |
| 6. Hypertension | | **No** | | | **Yes** | | | | | | | |  |  | | | |  | | |  |  |  |
|  |  | 0 | 24 | | | | | | | | | |  |  | | | |  | | |  |  |  |
| 7. Hypercholesterolemia | | **No** | | | | **Yes** | | | | | | |  |  | | | |  | | |  |  |  |
|  |  | 0 | 22 | | | | | | | | | |  |  | | | |  | | |  |  |  |

| **Variable** | | **Categories and Scores** | | | | | | | | | | | | | | | | | | | | | | | |  |  |
| --- | --- | --- | --- | --- | --- | --- | --- | --- | --- | --- | --- | --- | --- | --- | --- | --- | --- | --- | --- | --- | --- | --- | --- | --- | --- | --- | --- |
| 1. Sex | | **Female** | | | | | | **Male** | | | |  | | |  | | | |  | | | | | | |  |  |
|  |  | 0 | | | | | | 42 | | | |  | | |  | | | |  | | | | | | |  |  |
| 2. Age | | **40s** | | | | | **50s** | | | | | |  | | | |  | | |  | | | | | |  |  |
|  |  | 0 | | | | | 100 | | | | | |  | | | |  | | |  | | | | | |  |  |
| 3. Abdominal obesity | | **No** | | **Yes** | | | | | | |  | | | |  | | | |  | | | | | | |  |  |
|  |  | 0 | | 6 | | | | | | |  | | | |  | | | |  | | | | | | |  |  |
| 4. Obesity (BMI) | | **<23 kg/m^2^** | | | | | | | **<25 kg/m^2^** | | | | | **≥25 kg/m^2^** | | | | | | | | |  | |  | | |
|  |  | 0 | | | | | | | 15 | | | | | | | 20 | | | | |  | | |  | | |  |
| 5. Smoking | | **None** | | | **Light** | | | | | | **Moderate** | | | | | | | **Heavy** | | | |  | | | |  |  |
|  |  | 0 | 16 | | | | | | | 24 | | | | | | | | 16 | | | |  | | | |  |  |
| 6. Alcohol drinking | | **None** | | | **Normal** | | | | | | | **Heavy** | | |  | | | |  | | | | | | |  |  |
|  |  | 0 | 12 | | | | | | | | | 32 | | |  | | | |  | | | | | | |  |  |
| 7. Physical activity (METs) | | **≥1500** | | | | **<1500** | | | | | | **<1000** | | | **<500** | | | |  | | | | | | |  |  |
|  |  | 0 | 4 | | | | | | | | | 14 | | | 19 | | | |  | | | | | | |  |  |
| 8. Hypertension | | **No** | | **Yes** | | | | | | |  | | | |  | | | |  | | | | | | |  |  |
|  |  | 0 | | 17 | | | | | | |  | | | |  | | | |  | | | | | | |  |  |
| 9. Hypercholesterolemia | | **No** | | **Yes** | | | | | | |  | | | |  | | | |  | | | | | | |  |  |
|  |  | 0 | | 4 | | | | | | |  | | | |  | | | |  | | | | | | |  |  |
| 10. Diabetes Mellitus | | **No** | | **Yes** | | | | | | |  | | | |  | | | |  | | | | | | |  |  |
|  |  | 0 | | 23 | | | | | | |  | | | |  | | | |  | | | | | | |  |  |

**S8 Table.** Scores for each risk factor category for age 40–59 group

| **Variable** | | **Categories and Scores** | | | | | | | | | | | | | | | | | | | | | | | |  |  |
| --- | --- | --- | --- | --- | --- | --- | --- | --- | --- | --- | --- | --- | --- | --- | --- | --- | --- | --- | --- | --- | --- | --- | --- | --- | --- | --- | --- |
| 1. Sex | | **Female** | | | | | | **Male** | | | |  | | |  | | | |  | | | | | | |  |  |
|  |  | 0 | | | | | | 100 | | | |  | | |  | | | |  | | | | | | |  |  |
| 2. Age | | **60s** | | | | | **≥70** | | | | | |  | | | |  | | |  | | | | | |  |  |
|  |  | 0 | | | | | 69 | | | | | |  | | | |  | | |  | | | | | |  |  |
| 3. Abdominal obesity | | **No** | | **Yes** | | | | | | |  | | | |  | | | |  | | | | | | |  |  |
|  |  | 0 | | 20 | | | | | | |  | | | |  | | | |  | | | | | | |  |  |
| 4. Obesity (BMI) | | **<23 kg/m^2^** | | | | | | | **<25 kg/m^2^** | | | | | **≥25 kg/m^2^** | | | | | | | | |  | |  | | |
|  |  | 6 | | | | | | | 0 | | | | | | | 16 | | | | |  | | |  | | |  |
| 5. Smoking | | **None** | | | **Light** | | | | | | **Moderate** | | | | | | | **Heavy** | | | |  | | | |  |  |
|  |  | 0 | 24 | | | | | | | 29 | | | | | | | | 12 | | | |  | | | |  |  |
| 6. Alcohol drinking | | **None** | | | **Normal** | | | | | | | **Heavy** | | |  | | | |  | | | | | | |  |  |
|  |  | 0 | 22 | | | | | | | | | 46 | | |  | | | |  | | | | | | |  |  |
| 7. Physical activity (METs) | | **≥1500** | | | | **<1500** | | | | | | **<1000** | | | **<500** | | | |  | | | | | | |  |  |
|  |  | 0 | 27 | | | | | | | | | 21 | | | 22 | | | |  | | | | | | |  |  |
| 8. Abnormal liver function | | **No** | | **Yes** | | | | | | |  | | | |  | | | |  | | | | | | |  |  |
|  |  | 0 | | 24 | | | | | | |  | | | |  | | | |  | | | | | | |  |  |
| 9. Hypertension | | **No** | | **Yes** | | | | | | |  | | | |  | | | |  | | | | | | |  |  |
|  |  | 0 | | 31 | | | | | | |  | | | |  | | | |  | | | | | | |  |  |
| 10. Hypercholesterolemia | | **No** | | **Yes** | | | | | | |  | | | |  | | | |  | | | | | | |  |  |
|  |  | 13 | | 0 | | | | | | |  | | | |  | | | |  | | | | | | |  |  |
| 11. Diabetes Mellitus | | **No** | | **Yes** | | | | | | |  | | | |  | | | |  | | | | | | |  |  |
|  |  | 0 | | 40 | | | | | | |  | | | |  | | | |  | | | | | | |  |  |

**S9 Table.** Scores for each risk factor category for age ≥60 group

**S10 Table.** Summary statistics of risk scores

|  | Total scores  (Mean±SD) | 10-year Incidence probability  (Mean±SD) |
| --- | --- | --- |
| Age 20-39 | 160±48.24 | 0.12±0.08 |
| Age 40-59 | 93±62 | 0.69±0.37 |
| Age ≥ 60 | 111±65.52 | 2.09±0.78 |

**S1 Fig.** LASSO coefficient profiles for the model for age 20–39 group


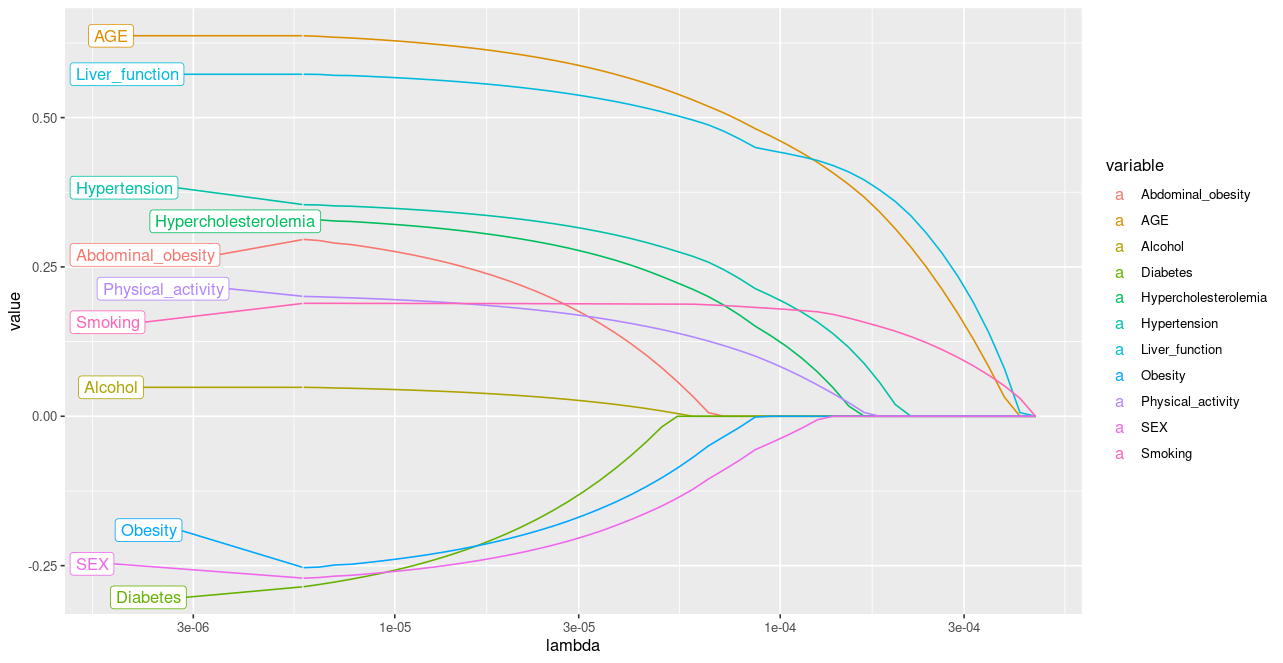


**S2 Fig.** LASSO coefficient profiles for the model for age 40–59 group


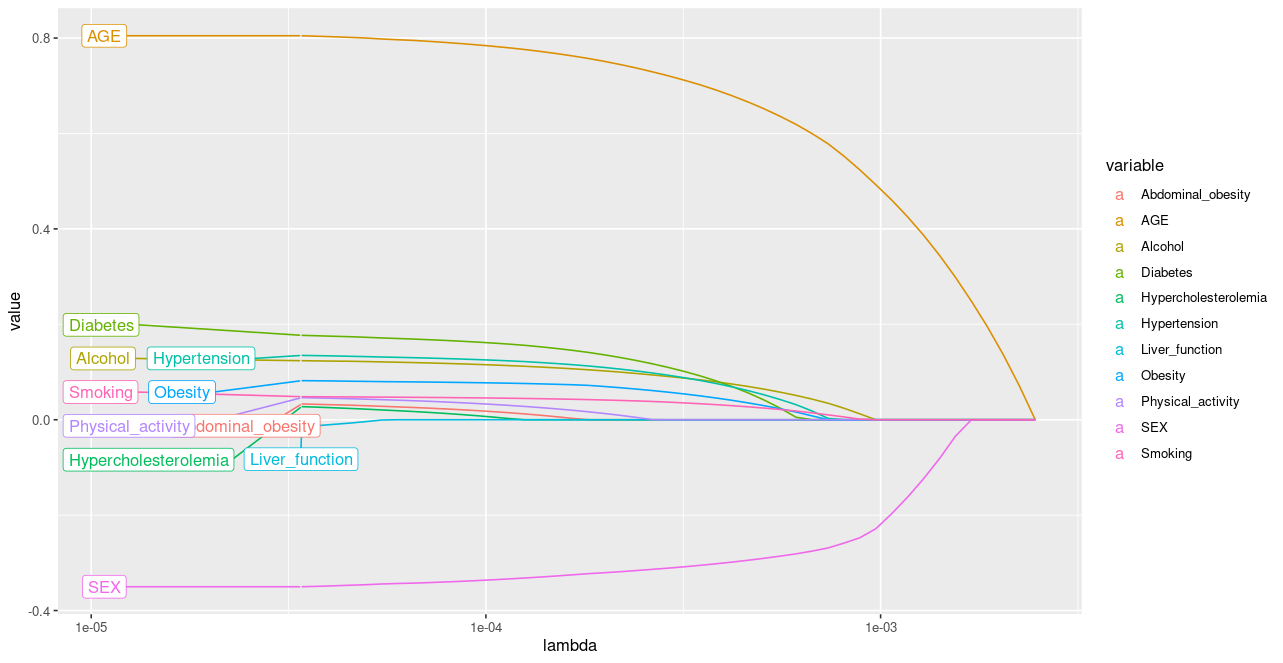


**S3 Fig.** LASSO coefficient profiles for the model for age ≥60 group

**
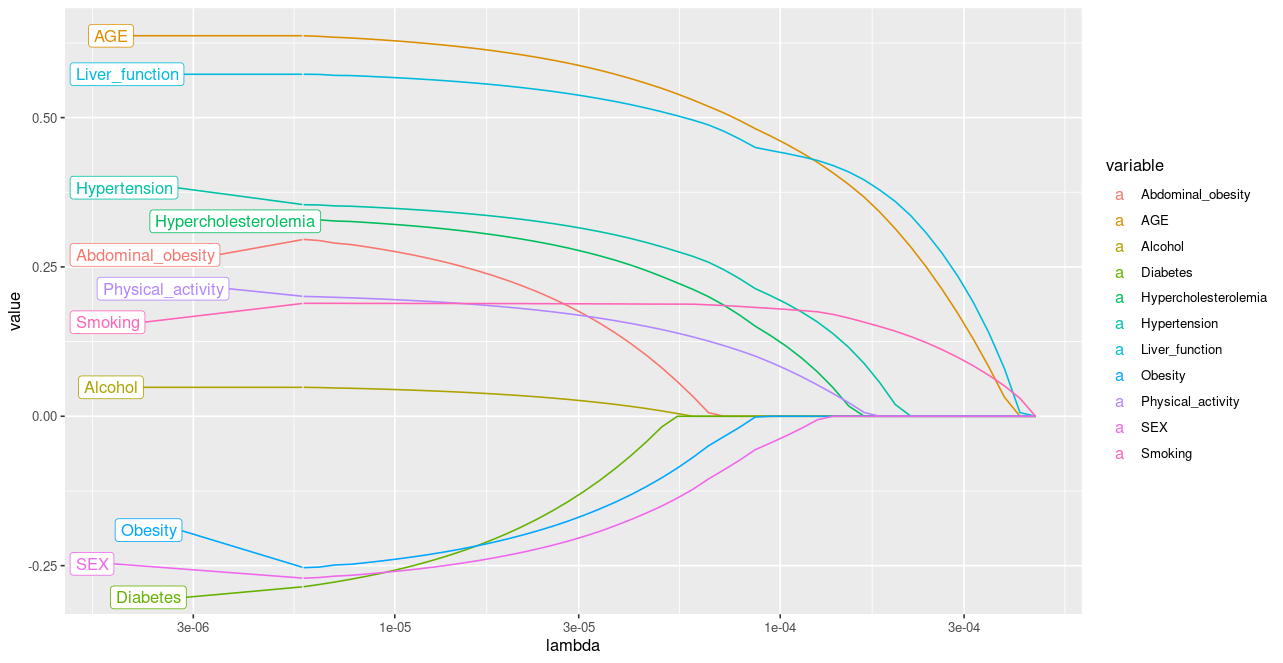
**

**Supplementary references**

1. Imperiale, T. F., Wagner, D. R., Lin, C. Y., Larkin, G. N., Rogge, J. D., & Ransohoff, D. F. Using risk for advanced proximal colonic neoplasia to tailor endoscopic screening for colorectal cancer. Annals of internal medicine. 2003;139(12):959-965.

2. Ma, E., Sasazuki, S., Iwasaki, M., Sawada, N., Inoue, M., & Shoichiro Tsugane for the Japan Public Health Center-based Prospective Study Group. 10-Year risk of colorectal cancer: development and validation of a prediction model in middle-aged Japanese men. Cancer epidemiology. 2010;34(5):534-541.

3. Freedman, A. N. et al., Colorectal cancer risk prediction tool for white men and women without known susceptibility. Journal of clinical oncology. 2009;27(5):686.

4. Driver, J. A., Gaziano, J. M., Gelber, R. P., Lee, I. M., Buring, J. E., & Kurth, T. Development of a risk score for colorectal cancer in men. The American journal of medicine.2007;120(3):257-263.

5. Steffen, A., MacInnis, R. J., Joshy, G., Giles, G. G., Banks, E., & Roder, D. Development and validation of a risk score predicting risk of colorectal cancer. Cancer Epidemiology, Biomarkers & Prevention,2014;23(11):2543-2552.

6. Wu, W. M., Gu, K., Yang, Y. H., Bao, P. P., Gong, Y. M., Shi, Y., ... & Fu, C. Improved risk scoring systems for colorectal cancer screening in Shanghai, China.Cancer Medicine. 2022;11(9):1972-1983.

7. Jeon JiHyoun, J. J., Du MengMeng, D. M., Schoen, R. E., Hoffmeister, M., Newcomb, P. A., Berndt, S. I., ... & Cao Yin, C. Y. Determining risk of colorectal cancer and starting age of screening based on lifestyle, environmental, and genetic factors. Gastroenterology. 2018;154(8):2152-2164.

8. Carr, P. R., Weigl, K., Edelmann, D., Jansen, L., Chang-Claude, J., Brenner, H., & Hoffmeister, M. Estimation of absolute risk of colorectal cancer based on healthy lifestyle, genetic risk, and colonoscopy status in a population-based study. Gastroenterology. 2020;159(1):129-138.

**Supplementary legends**

**S1 Table**. Previous research on risk scoring for colorectal cancer

**S2 Table.** Health checkup questionnaire in general health examination

**S3 Table.** Demographic and clinical characteristics of each age group

**S4 Table.** Cox Regression Model for 10-year Incidence of CRC for age 20–39 group

**S5 Table.** Cox Regression Model for 10-year Incidence of CRC for age 40–59 group

**S6 Table.** Cox Regression Model for 10-year Incidence of CRC for age ≥60 group

**S7 Table.** Scores for each risk factor category for age 20–39 group

**S8 Table.** Scores for each risk factor category for age 40–59 group

**S9 Table.** Scores for each risk factor category for age ≥60 group

**S10 Table.** Summary statistics of risk scores

**S1 Fig.** LASSO coefficient profiles for the model for age 20–39 group

**S2 Fig.** LASSO coefficient profiles for the model for age 40–59 group

**S3 Fig.** LASSO coefficient profiles for the model for age ≥60 group
